# 968. Methods to Increase Usability of Revised Scar Goniometry

**DOI:** 10.1093/jbcr/irag033.565

**Published:** 2026-04-06

**Authors:** Lisa R Kittleson, Sydney D Gudvangen

**Affiliations:** Regions Hospital Burn Center, Saint Paul, MN; Anders Group, Savage, MN

## Abstract

**Introduction:**

Standard goniometry does not consider how range of motion (ROM) in burn survivors is impacted by scar contracture. Previous research highlights the need for revised goniometric methods that consider cutaneous function, but implementation is limited by unfamiliarity with revised positions, lack of validation of specific positions, and inefficiency of Electronic Medical Record (EMR) documentation options.

**Methods:**

To address these limitations, a task force of burn rehab therapists in a verified burn center was established. After movement analysis as it relates to scar contracture and cutaneous functional units (CFUs), the task force created agreed-upon revised positions for ROM measurements at joints not previously validated. Next, photos and descriptions of revised positions were paired with those previously validated to create a comprehensive toolkit covering all measurable joints of the burn survivor. The toolkit was placed alongside goniometers in all patient rooms to increase access and consistency across providers. For updated data collection in the EMR, a ROM flowsheet was created that differentiated between standard and revised goniometric data. The photos for each revised position were uploaded into this flowsheet as a reference tool. The rehab discharge note template was revised to allow flowsheet data to be presented in an easily read chart format. Finally, as a reference tool for therapy providers outside of the burn center, a dot phrase was created to import photos of revised ROM positions into the discharge note along with a link to published research on scar goniometry.

**Results:**

By prioritizing the inclusion of scar goniometry into practice, described changes have promoted increased efficiency and communication for accurate data collection for a complex burn injury. References to scar goniometry are readily available to therapists, documentation has been streamlined, and communication regarding scar goniometry has improved.

**Conclusions:**

Readily available reference tools and customization of the EMR that reflect the rehab complexities of the burn survivor can positively impact the practical application of the principles of scar goniometry.

**Applicability of Research to Practice:**

Beyond recognizing the need for scar goniometry, efforts to increase uptake, promote consistency, incorporate tools, and enhance documentation can bridge the gap between research and everyday practice.

**Funding for the study:**

N/A.

